# The Book World of Medicine and Science

**Published:** 1896-10-03

**Authors:** 


### THE BOOK WORLD OF MEDICINE AND SCIENCE.

Twentieth - Century Practice of Modern Medical Science. Edited by Thomas L. Stedman, M.D. Volume IV. "Diseases of the Vascular System and Thyroid Gland." (London: Sampson Low, Marston, and Co. 1896.)

More than half of this volume is occupied by a treatise, of a systematic rather than a clinical nature, upon diseases of the heart, by Dr. James T. Whittaker, of Cincinnati; the rest of the space being divided between Dr. A. E. Sansom, of London, on "Diseases of the Blood Vessels"; Dr. Bertrand Dawson, of London, on "Diseases of the Lymphatic Vessels"; and Dr. George R. Murray, of Newcastle-on-Tyne, on "Diseases of the Thyroid Gland."

Dr. Whittaker has evidently spent a very large amount of labour in producing what certainly is a full and fairly complete account of the diseases of the heart. We cannot say, however, that the result is altgether satisfactory. The arrangement is too artificial, the attempt to isolate and describe separately morbid conditions which, as they usually occur, are much entangled, gives to many of its paragraphs an air of theory rather than of practice, while the constant quotation of the often opposed opinions of other authors, gives to the treatise a stronger flavour of the study than of the hospital ward and post-mortem room. Yet with all this we miss any such general description of the physical and physiological problems involved in disorders of the heart as one might expect in a work of such dimensions.

The most interesting parts of Dr. Sansom's article are those which deal with atheroma of the coronary arteries, aneurism of the aorta, and arteriosclerosis.

In the treatment of atheroma of the coronary arteries interfering with the nutrition of the heart, Dr. Sansom lays emphasis on the necessity of rest and milk diet, the latter being given, not as a low diet, but as one which does not irritate the arterioles and thus increase tension by producing complex albuminoids during its assimilation.

Iodide of sodium is recommended as a means of keeping down arterial tension, and it is pointed out that such treatment may, with due intermissions, be carried on for months, and even years.

In regard to the treatment of aortic aneurism by rest and diet, Dr. Sansom would not continue it so long as is sometimes done. He says "the very protracted treatment by recumbency is a grievous burden, often greatly resented. Two months of such treatment will induce consolidation within the sac if such consolidation is within the possibilities of therapeutics." We are not sure, however, that many people suffering from aneurism would not prefer a still longer continuance of recumbency, even as a mere palliative method, to undergoing the surgical proceedings suggested on its failure to produce consolidation.

Dr. Bertrand Dawson's article on "Diseases of the Lymphatic Vessels" will well repay reading, although many of the conditions dealt with are of comparative rarity, and the section by Dr. George Murray on "Diseases of the Thyroid Gland," including exophthalmic goitre, is full of interest.

Obesity; Its Causes and Treatment. By Thomas Dutton, M.D. (London: Henry Knight, 1896. 1s. 6d.)

The author of this little work has written several treatises of a similar nature on "The Rearing and Feeding of Children," "Sea Sickness," "Health Resorts," and "Domestic Hygiene," subjects which obviously appeal to the public appetite for things medical. The present volume is addressed to "medical men and others." We cannot help thinking that the first moiety of its intended readers will find a deficiency of scientific authority in the handling of this difficult subject; the words of the author carry no conviction with them, and there is a want of physiological knowledge as obvious as is the want of literary style. In one respect, however, we are in entire agreement with the author; our disapproval of quacks and their nostrums is as great or greater than his. In classifying obesity Dr. Dutton divides the condition into two classes, hereditary and acquired. The former, he says, is not a common disease. Here we think the author makes a mistake. It is the hereditary tendency to put on fat which runs through many families which is the most frequently met with and most difficult to treat. The strictly "acquired" form is far easier to control, less dangerous to reduce, and of less common occurrence. The "man in the street" who is interested in his own ailments and suffers from an excessive deposition of fat will read these few pages without danger to himself, nay, more, if he assimilates the contents he will benefit to the extent that he will not treat his own symptom but submit his vile body to a legitimate practitioner, or a medical specialist on obesity.

Galvanism in the Treatment of Neuritis and Other Diseases. By Carey Coombs, M.D.Lond. (Bristol: John Wright and Co. 1896, pp. 54. Price 1s.)

It is not very easy to find good and sufficient reason for the publication of this little book, for most of the information it contains can easily be got elsewhere. Nevertheless, it gives a fair sketch, so far as its space permits, of the clinical uses of electricity in medical practice. There are, however, some points in regard to which a larger personal experience would probably have led the author to express himself more cautiously. In addressing a Medical Society on the various uses of electricity in the treatment of disease—a thing far too much overlooked by many general practitioners—we think that the author of this book did well. Whether he did well in publishing his book in the form of a manual of galvanism is another matter. It is too small to be really explanatory to those who have no acquaintance with the subject, while it is far too elementary to be of interest to others.

